# The resilience of Jewish communities living in the diaspora: a scoping review

**DOI:** 10.3389/fpsyg.2023.1215404

**Published:** 2023-08-16

**Authors:** Judith E. M. Meijer, Anja Machielse, Geert E. Smid, Winnie Schats, Miek C. Jong

**Affiliations:** ^1^Department Humanism and Social Resilience, University of Humanistic Studies, Utrecht, Netherlands; ^2^ARQ National Psychotrauma Center, Diemen, Netherlands; ^3^Scientific Information Service, Netherlands Cancer Institute, Amsterdam, Netherlands; ^4^Department of Health Sciences, Mid Sweden University, Sundsvall, Sweden; ^5^Department of Community Medicine, Faculty of Health Sciences, National Research Center in Complementary and Alternative Medicine (NAFKAM), Arctic University of Norway (UiT), Tromsø, Norway

**Keywords:** adaption, adversity, community resilience, diaspora, Jewish community, migration

## Abstract

**Introduction:**

Throughout history, Jewish communities have been exposed to collectively experienced traumatic events. Little is known about the role that the community plays in the impact of these traumatic events on Jewish diaspora people. This scoping review aims to map the concepts of the resilience of Jewish communities in the diaspora and to identify factors that influence this resilience.

**Methods:**

We followed the Joanna Briggs Institute (JBI) methodology. Database searches yielded 2,564 articles. Sixteen met all inclusion criteria. The analysis was guided by eight review questions.

**Results:**

Community resilience of the Jewish diaspora was often described in terms of coping with disaster and struggling with acculturation. A clear definition of community resilience of the Jewish diaspora was lacking. Social and religious factors, strong organizations, education, and communication increased community resilience. Barriers to the resilience of Jewish communities in the diaspora included the interaction with the hosting country and other communities, characteristics of the community itself, and psychological and cultural issues.

**Discussion:**

Key gaps in the literature included the absence of quantitative measures of community resilience and the lack of descriptions of how community resilience affects individuals’ health-related quality of life. Future studies on the interaction between community resilience and health-related individual resilience are warranted.

## 1. Introduction

“A sustainable human community must be designed in such a manner that its ways of life, its businesses, its economy, physical structures, technologies, and social institutions, do not interfere with nature’s inherent ability to sustain life” (Capra and Luisi, 2014).

Jewish communities worldwide have a long and continuous history of traumatic events. The adaptation of Jewish individuals to these traumatic events, especially the Holocaust, has been well documented (Stein, 2009; Keysar, 2014; Lurie, 2017; Diamond et al., 2020; Zimmermann and Forstmeier, 2020). However, research institutions such as the Institute for Jewish Policy Research have mostly reported on community resilience in Israel (Institute for Jewish Policy Research, 2015; Dellapergola and Staetsky, 2020). This paper is based on the assumption that it is also important to understand how the Jewish communities affect the resilience, life, and health of Jewish people living in the diaspora outside Israel.

In this paper we define Jewish diaspora communities as the core and enlarged Jewish population, as described by the Institute for Jewish Policy Research (Institute for Jewish Policy Research, 2015). According to recent numbers (The Jewish Agency for Israel, 2022) 8.25 million Jews live outside Israel (55% of the 15.3 million Jews worldwide). Most of them live in the United States (6 million). Other countries hosting Jewish diaspora communities are France (446.000), Canada (393.500), Great Britain (292.000), Argentina (175.000), Russia (150.000), Germany (118.000), Australia (118.000) or smaller communities, such as the Netherlands (30.000–50.000) (Van Solinge and de Vries, 2001; Van Solinge et al., 2010). Around 38 countries worldwide have a Jewish population of 500 people or fewer.

The concept of resilience has been applied in many scientific disciplines and is one of the core concepts in contemporary social studies (Van der Schoor et al., 2021; Derakhshan et al., 2022). Resilience is often studied from an individual perspective, even though many of these studies have stressed the relevance of the social dimension to maintain and strengthen people’s resilience: the role of the family, the neighborhood, and the community (Bronfenbrenner et al., 1986; van der Kolk, 2014).

In the research literature, three perspectives on resilience can be distinguished (Pfefferbaum et al., 2015; Verbena et al., 2021). First, the resource-based perspective focuses on certain core attributes and resources that resilient entities possess (Tengblad and Oudhuis, 2018). Second, the outcome-based perspective focuses on positive outcomes amidst adversity (Bonanno, 2004; Bonanno et al., 2015). Last, the process-based perspective explores the working mechanisms involved in navigating using resources in the context of adversity to achieve a positive outcome (Norris et al., 2008). This ‘fluctuating’ process is described as a phenomenon occurring through interactions within and between multiple levels, i.e., individual, community, and society (Infurna and Luthar, 2018; Wiles et al., 2019).

In recent decades, there is growing interest in the meaning of the community for individual resilience (Hobfoll et al., 2007; van der Kolk, 2014) and the need for a better understanding of the concept of community resilience. It has been reported that contextual factors, such as the family or the community in which people live, are likely to exert more influence on individual resilience outcomes than individual traits (Landau, 2007; Ungar, 2011; Fischer and McKee, 2017). Several authors have highlighted the relevance to gain a better understanding of the dynamics between community resilience and individual resilience (Norris et al., 2008). Others study the impact of traumatic events on the resilience and health of individuals (Duckers et al., 2017; South et al., 2018) as there is growing evidence of a relationship between somatic and psychological conditions, social context, well-being, and functioning (Scott et al., 2013; Huber, 2014).

A systematic review of community resilience concluded that community resilience remains an amorphous concept that is understood and applied differently by different research groups (Patel et al., 2017). Depending on the perspective of the author, community resilience is, respectively, seen as a continuous adaption process to adversity, the absence of negative effects, the presence of various positive factors, or a combination of all three. Although the definitions differ, Patel et al. (2017) found nine main categories which were subdivided into nineteen sub-elements of community resilience that were common among the definitions in the reviewed scientific articles on disasters. The main elements they mentioned were local knowledge, community networks and relationships, communication, health, governance and leadership, resources, economic investment, preparedness, and mental outlook. Patel et al. (2017) suggest for future research to focus on these main elements as they can be measured and improved, which may contribute to understanding as well as policy making.

While the review of Patel et al. concentrated on the literature on disasters, Flora et al. (2016) focused their research on the dynamic process of communities constantly changing and responding to adversities. Based on that, they developed a theoretical model, the Community Capital Framework, that consists of seven resource categories or ‘capitals’: three material capitals – natural, built, and financial – and four immaterial capitals – human, social, cultural, and political. Van der Schoor (2020)^1^ assumes these resources can function in several ways, (1) as a buffer against the impact of adversity, (2) as compensation for the negative effects of the adversity and (3) as a catalyst of community members’ ability to change or transform the structural conditions of adversity. Comparable models, in which several capitals are defined can be found in studies aiming to measure community resilience (Mayunga, 2009; Derakhshan et al., 2022).

Though some research has been conducted on the resilience of diaspora communities (Kidron, 2012; Smid et al., 2018; Alefaio-Tugia et al., 2019), there is currently no published systematic overview on the factors that hinder or strengthen the resilience of communities living in the diaspora. Therefore, we aimed to conduct a systematic review of the existing literature on the resilience of Jewish communities living in the diaspora to identify factors that influence community resilience. A scoping review was considered the most suitable type of systematic review method for this purpose, as it is a form of knowledge synthesis that addresses exploratory research questions aimed at mapping key concepts, identifying key characteristics related to these concepts, examining how research is conducted on that topic, and identifying knowledge gaps (Colquhoun et al., 2014). The current scoping review intends to inform researchers on how to design future studies on drivers and barriers of diasporic community resilience and inform policymakers, leaders, and members of communities on methods to strengthen the resilience of Jewish diasporic communities outside Israel. A preliminary search for existing reviews on this topic was conducted in PROSPERO, MEDLINE, databases of the Joanna Briggs Institute, and the Cochrane Database of Systematic Reviews. No protocols for a similar scoping review were identified.

## 2. Method

The predefined protocol for this scoping review was published in the public domain (Meijer et al., 2022). The protocol followed the Joanna Briggs Institute (JBI) Reviewers’ Manual for scoping reviews (Aromataris et al., 2020; Jong et al., 2021) and guidance for conducting systematic scoping reviews as published by Peters et al. (2020). Results are reported according to the Preferred Reporting Items for Systematic Review and Meta-Analyses extension for Scoping Reviews (PRISMA-ScR) checklist (Tricco et al., 2018; Supplementary File 1). Since a scoping review analyzes data already published in the literature, no ethical review was needed.

### 2.1. Eligibility criteria

To identify and define the main concepts in the review questions the Participants/Concept/Context (PCC) framework was used (Peters et al., 2021). The PCC framework is recommended by JBI as a guide to formulate the main review questions of the scoping review.

### 2.2. Participants

We included studies about Jewish communities worldwide that describe Jewish persons aged 18 years and older. We defined Jewish communities as the core and enlarged Jewish population, as described by the Institute for Jewish Policy Research (Institute for Jewish Policy Research, 2015). The core Jewish population includes people who self-identify as Jewish in social surveys and do not have another monotheistic religion. It also includes people who may not recognize themselves as Jewish but have Jewish parents and have not adopted a different religious identity. It further consists of all converts to Judaism by any procedure and other people who declare themselves Jewish, even without having undergone conversion. The enlarged Jewish population includes the sum of (a) the core Jewish population; (b) all other people of Jewish parentage who, by core Jewish population criteria, are not currently Jewish (e.g., they have adopted another religion or otherwise opted out); and (c) all respective non-Jewish household members (spouses, children, etc.).

### 2.3. Concept

Resilience is defined as the ability to withstand adversity and the capacity to bounce back from potentially traumatic events (Vanhamel et al., 2021). Articles on closely related concepts, such as coping, recovery, dealing with adversity, adaptation, and acculturation were included in the review. Articles included were not limited to resilience in relation to health or healthcare. Articles describing individual resilience only, without mentioning a link to the community or contextual variables, were excluded. Articles that describe the resilience of Jewish communities in relation to historical events before the Second World War were also excluded.

### 2.4. Context

Diasporic communities are defined as communities of people who live outside their shared country of origin or ancestry but maintain passive or active connections with it (Nazeer, 2015). A diaspora includes both emigrants and their descendants. In this scoping review, Israel is not considered a diaspora; therefore, studies on Jewish communities in Israel were excluded.

### 2.5. Objectives

The review addressed the following questions:

Review question 1. What concepts of resilience are being described for Jewish communities living in the diaspora? How is resilience defined, and which underlying theoretical models have been used to understand how the resilience of Jewish diasporic communities functions?

Review question 2. Which trauma or underlying causes of stress (e.g., Holocaust, genocides, or racism) are described in relation to the resilience of Jewish communities living in the diaspora?

Review question 3. Which facilitating factors for the resilience of Jewish communities in the diaspora are described?

Review question 4. Which barriers to the resilience of Jewish communities in the diaspora are described?

Review question 5. What methods are used in studies to measure the resilience of communities?

Review question 6. What is described about the relationship between the resilience of Jewish diasporic communities and the health-related quality of life of individuals belonging to the community?

Review question 7. What are the key gaps in the literature on the resilience of Jewish diasporic communities?

Review question 8. Are there any ethical issues or challenges identified that relate to the resilience of Jewish diasporic communities?

### 2.6. Types of sources

The selected studies describe peer-reviewed scientific research publications on quantitative and qualitative methodologies, including randomized controlled trials, controlled (non-randomized) clinical trials, controlled before-after studies, prospective and retrospective comparative clinical studies, non-controlled prospective and retrospective observational studies, cohort studies with before-after design, case series, case reports, qualitative studies, PhD theses, systematic reviews, meta-analyses, meta-syntheses, narrative reviews, mixed-methods reviews, qualitative reviews, and rapid reviews. Studies published as master’s or bachelor’s theses, information from books or book chapters, analyses/reviews of books, and analyses/reviews of movies were excluded because we considered them to be outside the scope of the peer-reviewed scientific literature. Conference reports/proceedings were also excluded because they may not contain adequate detailed information for our scoping review and/or describe preliminary data.

### 2.7. Search strategy and study selection

Two information specialists developed the search strategy (Supplementary File 2) aiming to locate studies already published in the literature. Medical Subject Headings (MeSH) (or comparable controlled vocabularies) and free text terms (terms in the title and/or abstract of the articles) were used in databases with controlled vocabulary. The search strategy was adapted broadly in databases without controlled vocabularies to obtain the maximum search yield. In addition to the database searches, the reference lists of articles selected for full-text review were screened for additional studies. In the searches, no restrictions were applied to the study design, date, or language. However, only articles published in English, Danish, Dutch, German, Hebrew, Norwegian, and Swedish were included. Articles published after World War II to the present were included. Excluded were articles describing the resilience of Jewish communities in relation to earlier historical events.

The same information specialists performed searches from April 25 to 28, 2021 in the following databases: PsycInfo, Ovid Medline ALL, Embase, PTSDpubs, SSRN, Sociological Abstracts, JPR, Berman, Rambi, NARCIS, Google Scholar, and Web of Science. Additionally, the gray literature was searched to identify possible relevant PhD theses for inclusion in this scoping review (Paez, 2017). The source of the gray literature search was OpenGrey.eu.

Following the literature search, all identified records and citation abstracts were collated and uploaded into the review web tool Rayyan to facilitate the study selection and data extraction process. Duplicates were removed. Each step in the scoping review process (screening, study inclusion, data extraction etc) was performed independently by at least two authors. The search results and decisions regarding inclusion/exclusion were recorded in Rayyan and are reported in the Prisma-ScR flow diagram (Tricco et al., 2018).

### 2.8. Data extraction

A pilot data extraction of two articles (Azoulay and Sanchez, 2000; Chalew, 2007) was carried out in Rayyan. After piloting, the authors extracted and assessed the data in relation to the scoping review questions. Accordingly, two items were added to the data extraction form (Supplementary File 3).

### 2.9. Collating and summarizing the results

All authors were involved in the process of data synthesis and interpretation. A summary table with detailed information about every included article was provided (see Table 1).

**Table 1:** Characteristics of included studies.

Author	Year	Study design	Disciplinary field	Study aim	Methods	Sample (size)	Setting	Background	Adversity
Aronson, J.; Boxer, M.; Brookner, M. A.; Magidin de Kramer, R.; Saxe, L.	2020	Cross-sectional study	Epidemiology	To understand the impact of COVID-19 on the financial well-being and emotional health of members of the Jewish community, the role Jewish institutions, and the value of online Jewish life and Judaism.	Survey	1,325 respondents	USA	Respondents, who considered themselves Jewish and lived in Baltimore area	COVID-19
Azoulay, B.; Sanchez, W.	2000	Case study	Counseling psychology	To discuss treatment issues and challenges facing mental health counselors pertaining to the integration of culture in working with Israeli clients and families. To provide basic information (key issues and concepts) to work with Israeli families who immigrate to the United States	Descriptive analysis	1 family	USA	Israeli	(double) migration
Carp, J. M.	2007	Case study	Policy science	To describe the experience of the Chicago Jewish community in its preparation for a second responder strategy in case of critical incidents	Descriptive analysis	The Chicago Jewish community	USA	Not specified	Traumatic events in the broad sense
Chalew, G. N.	2007	Case study	Sociology	To describe the way Jewish leadership rebuilds their community after Hurricane Katrina in New Orleans	Descriptive analysis	The New Orleans Jewish community	USA	Not specified	Natural disaster
Elo, M.; Vemuri, S.	2016	Case study	Economics	To study how resource employment and economic activity is organized within a diaspora. Post-Soviet era Bukharian Jewish diaspora is the subject of the case study for understanding the self-organization and forms of resource employment.	Ethnography, descriptive analysis	Bukharian communities	Worldwide	Bukharian	Migration
Frogel, E.	2015	Qualitative study	Psychology	To identify and clarify systems that influence acculturation processes of first- and second-generation Afghan Jewish (AJ) immigrants in the United States To increase multicultural awareness about immigrant populations in the U.S.	Ethnography, interviews, thematic analysis	six male and female first generation and six male and female second-generation AJ participants >18 years	USA	Afghan	Migration
Gidley, B., Kahn-Harris, K.	2012	Case study	Policy science/sociology	To understand how communal leadership has defined the community and used this to legitimate its authority.	Interviews, documentary analysis	17 Anglo-Jewish key figures	UK	Not specified	Not specified
Glöckner, O.	2007	Qualitative study	Philosophy	To analyze how Russian Speaking Jews (RSJ) professional and intellectual elites in Israel and Germany define themselves after resettlement, where do they see their scope of action presumed, they have managed to integrate into their host society but remained interested in RJ issues, to what extent RSJ elites are willing to play a leading role in RSJ community building, to find out whether RJ elites are attempting to build and maintain transnational ties.	Interviews, content analysis	70 RSJ elites, of which 35 in Germany	Germany	Russian	Migration
Heitlinger, A.	2009	Case study	Sociology	To understand what the reports of the reunions tell about the ways in which history, generation, ethnicity, migration, biography and memory intertwine, what can be learned about identity and community formation, and what do the reunions tell us about the selective use of memory as a strategy to engage with the past, the present and the future?	Descriptive analysis	12 Czech/SlovakJewish reunions	Several	Czech/Slovak	Migration
Horwitz, S.	2007	Case study	Psychology/trauma studies	To describe the strategic response by the UJA-Federation of New York to establish and fund the development of the Israel Trauma Coalition	Descriptive analysis	Jewish community in the USA and Israel	USA	Not specified	Not specified
Pirutinsky, S.; Cherniak, A. D.; Rosmarin, D. H.	2020	Cross-sectional study	Psychology/mental health	To assess the impact of the COVID-19 pandemic on members of the American Jewish community generally, and to explore specifically the relationship between COVID-19 exposure, religiosity, and distress.	Survey	419 respondents	USA	Orthodox/Haredi	COVID-19
Pollock, D. M.	2007	Text and opinion	Religious studies/trauma studies	To describe Jewish resilience in general	Descriptive analysis	Jewish community in general	Worldwide	Not specified	Not specified
Storr, V. H.; Haeffele-Balch, S.; Grube, L. E.	2007	Qualitative study	Sociology/economics	To explore the post-disaster social learning process by focusing on the importance of social capital in facilitating social learning after a disaster, including facilitating community members’ ability to communicate their desire to return, to assess damage, to overcome barriers to rebuilding through collective yet voluntary action, and to learn from and imitate others’ successes.	Interviews, documentary analysis	16 participants of the Orthodox Jewish community in the Rockaway Peninsula in New York	USA	Orthodox /Haredi	Natural disaster
Storr, V. H.; Grube, L. E.; Haeffele-Balch, S.	2017	Case study	Sociology	To contribute to existing research on the role of civil society post-disaster by considering the arguments around polycentric versus monocentric orders based on the assumption that a society that is more polycentric is more flexible and adaptable to changing needs of citizens.	Interviews, descriptive analysis	The Orthodox Jewish community in the Rockaway Peninsula in New York	USA	Orthodox/Haredi	Natural disaster
Vanhamel, J.; Meudec, M.; Van Landeghem, E.; Ronse, M.; Gryseels, C.; Reyniers, T.; Rotsaert, A.; Ddungu, C.; Manirankunda, L.; Katsuva, D.; Koen, P. G.; Nöstlinger, C.	2021	Qualitative study	Tropical medicine	To understand the impact of COVID-19 on Orthodox Jewish communities in Antwerp, Belgium, during the first ‘lockdown’ period in Belgium and to explore how local response mechanisms and coping strategies were shaped and developed from within these communities, building on pre-existing local resources and social networks	Interviews, thematic analysis	15 participants	Belgium	Orthodox/Haredi	COVID-19
Vollhardt, J. R.; Nair, R.	2018	Qualitative study	Psychology	To explored how members of diaspora groups with different histories of collective victimization experience its consequences within their communities	Focus groups, thematic analysis	16 Jewish participants	USA	Not specified	Not specified

### 2.10. Patient and public involvement

The first author was employed by the Dutch Jewish Welfare Organization from 2017 to 2021. Furthermore, the review questions of this scoping review were actively discussed and refined with input from three experts within the international Jewish community.

### 2.11. Deviations from the protocol

In the predefined protocol, it was described that the PAGER methodological framework is used to assist data analyses (Meijer et al., 2022). However, due to the large variety in designs, methodologies, populations, and outcomes of the sixteen studies included, it was not feasible to apply this framework. Instead, an inductive analysis in Atlas-ti was conducted to retrieve a thematic categorization of the mentioned positive factors and barriers (Kiger and Varpio, 2020). Next, all positive factors and barriers were deductively analyzed using the elements of Patel et al. (2017) and the seven capitals of the Community Capitals Framework of Flora et al. (2016) in order to gain further insight into the characteristics of the community resilience of the Jewish diaspora.

### 2.12. Article identification and selection process

**Figure 1:**
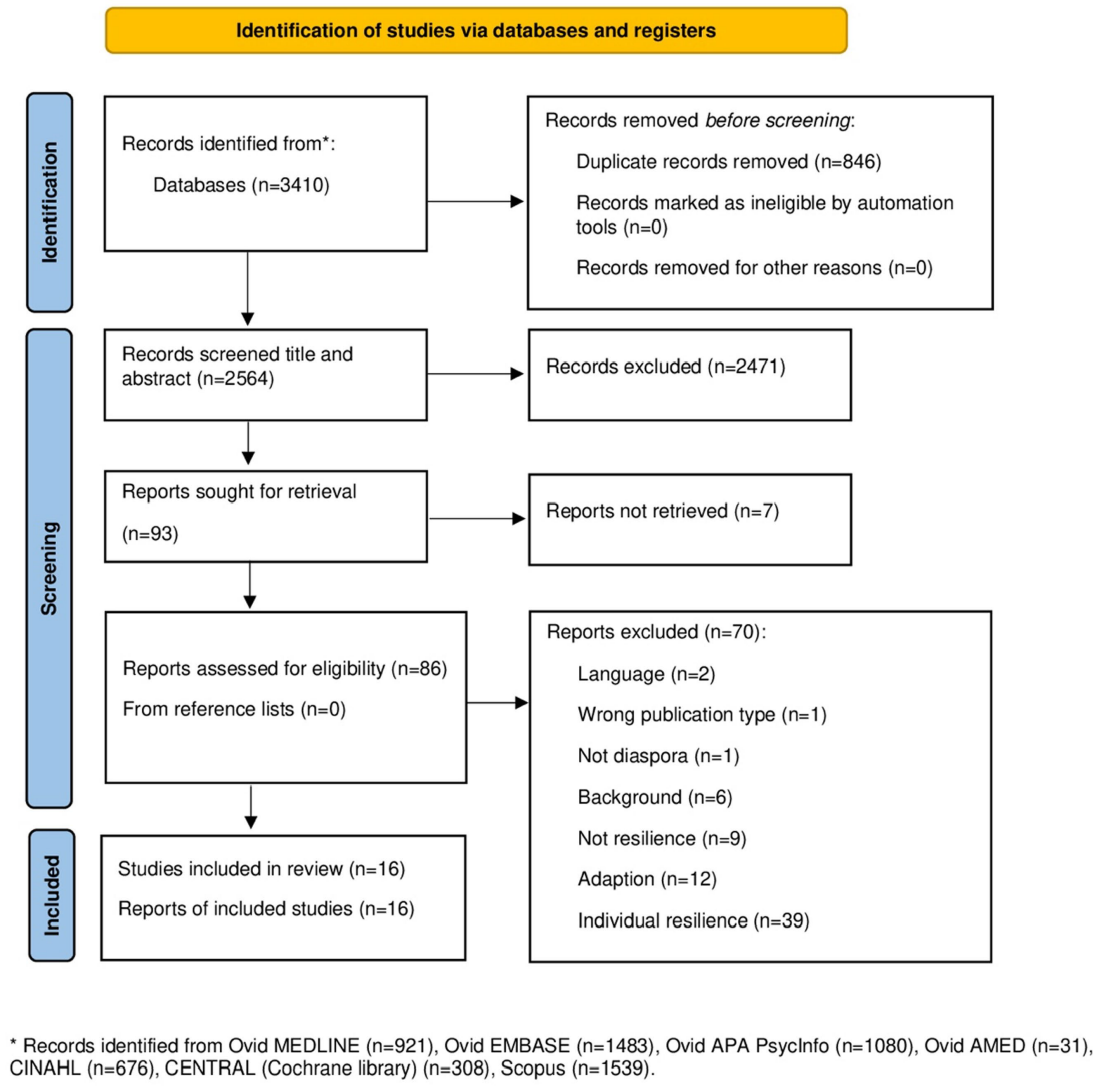
Prisma 2020 flow diagram scoping review.

The database and gray literature search yielded 2,564 records after deduplication (Figure 1).

Screening of titles and abstracts resulted in a first selection, after which 2,471 records were excluded because they did not meet the inclusion criteria. Of the resulting 93 records, seven records were not retrieved, of which six were dissertations. A total of 86 records were therefore assessed for eligibility. After the full-text screening, sixteen articles met the eligibility criteria for inclusion in the scoping review.

## 3. Results

### 3.1. Characteristics of included articles

A summary of the included articles and their study designs (*n* = 16) can be found in Table 1. Included articles were methodologically diverse, mostly based on case studies (*n* = 8). Other types of studies included were qualitative studies (*n* = 5); cross-sectional studies (*n* = 2), and text and opinion (*n* = 1). Nine of the 16 articles were based on empirical research. The research was conducted by a broad diversity of disciplinary fields including amongst others psychology, economics and sociology (see Table 1). The applied empirical methods were interviews (*n* = 6), surveys (*n* = 2) and one focus group. Several types of analysis were conducted: descriptive (*n* = 8), thematic (*n* = 3) documentation (*n* = 2), ethnographic (*n* = 2) and one content analysis. Two records were dissertations. Nine articles originated from the USA, and others were from Europe (*n* = 3) or reported on diasporic populations in more than one country (*n* = 4). The articles were either published from 2000 to 2010 (*n* = 7) or after 2010 (*n* = 9). In only five out of sixteen studies, the migration background of the Jewish diasporic community was specified.

### 3.2. Concepts of community resilience, definitions, and underlying theoretical models (review question 1)

The concepts of community resilience in the included articles are listed in Table 2. The main finding is that the reviewed articles use different definitions of resilience and various underlying theoretical models or frameworks for Jewish diaspora community resilience. In only one of the articles (Pollock, 2007) community resilience was defined as such. Most studies characterized the diasporic Jewish communities by means of a specific geographic location, for example the Antwerp community (*n* = 9). Other studies characterized the Jewish community based on their migration background, for example Russian Jewish migrants (*n* = 5). In two studies, the Jewish community was characterized by religious affiliation, in both cases as Orthodox communities. Furthermore, resilience was used as a concept close to other related concepts, such as coping, mitigation, survival, or recovery.

**Table 2:** Concepts of community resilience.

	Type community	Perspective on resilience	Concept of resilience described	Definition of resilience	Underlying theoretical model, including author
Aronson, J.; Boxer, M.; Brookner, M. A.; Magidin de Kramer, R.; Saxe, L.	Geographical	Resource-based	Financial wellbeing; coping and emotional health; relationship with Jewish organizations and synagogues; charitable support for Jewish organizations; online Jewish life	ND	ND
Azoulay, B.; Sanchez, W.	Migration background	Resource-based, process-based	Process of dynamic change	ND	ND
Carp, J. M.	Geographical	Process-based	Resiliency response models: preparedness and awareness, providing crisis intervention and trauma assistance; providing emergency assistance; assisting affected agencies in gaining control of the crisis; providing a command structure for a coordinated response; coordinating communications, is short- and long-term and involves individual, family, community, and institutional level. People use their own strength instead of falling to victimhood	ND	Donald J. Cohen and Irving B. Harris Center for Trauma and Disaster Intervention
Chalew, G. N.	Geographical	Outcome-based, process-based	Making the Jewish community in New Orleans stronger, more cohesive, committed, and revitalized, after a major event (adversity). Three phases: (1) Pikuach Nefesh: Saving Lives; (2) Return to home and Initial Recovery (3) Renaissance: Beyond recovery to transformation	ND	ND
Elo, M.; Vemuri, S.	Migration background	Resource-based	Self-organize human resource utilization and better employment of its inherent talent, and thus benefit both host and home countries. Ethnic cohesion and co-operation, self-organization, education, Bukhara Jewish support system; the community is built on its Jewish ontology and values and norm regulating the good ‘Bukharian Jew’-concept - > an identity related motivation mechanism for organization of collective bettermen and participation	ND	Positive organizational scholarship (POS) (Cameron and Caza, 2004) on organizing diaspora resources through various network organizations (Nohria and Eccles, 1992; Granovetter, 2005), and combining diaspora theories on resources (Tung, 2008) and resource-based view (RBV) (Barney, 2001a,b)
Frogel, E.	Migration background	Resource-based, process-based	Struggle with acculturation. A prevailing attitude and strength that acted as a protective factor for many Afghan Jewish immigrants of both generations. When faced with adversity, this attitude was helpful to them in persevering and moving forward with their life. The hopefulness, humor, lightheartedness, and strength indicative of this attitude helped them manage many hardships as well as personal struggles that were overcome by remaining strong in their positive attitude. Psychological acculturation includes behavioral changes such as dress and language, and acculturative stress such as anxiety and depression. Adaptation is both psychological and sociocultural (Berry, 2003).	ND	An ecological model perspective highlighting the systems level influence of acculturation and intergenerational experiences (Bronfenbrenner, 1994). Theory on acculturation (Kim and Abreu, 2001)
Gidley, B., Kahn-Harris, K.	Geographical	Process-based	Dealing with security/insecurity. A growing societal concern with security as part of the ‘risk society’ or ‘liquid modernity’. For Bauman, the ‘liquid modernity’ of our age means living ‘under the constant condition of anxiety’, prone to become ‘neurotic about matters of security’ (1992: 697). ‘Faith-based’ groups and identities being given an increasingly prominent role in social provision, education and policy, creating a powerful impetus for communal professionalization and self-reflection	ND	ND
Glöckner, O.	Migration background	Resource-based, process-based	Cultural self-assertion	ND	Keller’s model of strategic elites that emphasizes the existence and necessity of heterogeneous, pluralistic elites for modern societies -competing each other, but also interdependent and with specific functions (1963).
Heitlinger, A.	Migration background	Process-based	The four overlapping concepts, helpful in both addressing some of the issues and connecting them to the empirical evidence presented are autobiographical occasion, commemoration, reflective nostalgia and diaspora. Connection with Jewish people of the same background/same history, leading comparable lives, feeling like a family (after most original families were diminished or non-existent because of the holocaust)	ND	ND
Horwitz, S.	Geographical	Process-based	Model of disaster planning and emergency response, Prevention of distress	ND	ND
Pirutinsky, S.; Cherniak, A. D.; Rosmarin, D. H.	Religious	Resource-based	Religiosity as a factor in coping with COVID-19 exposure and distress / utilizing positive religious coping during the pandemic as a means of providing mental health benefits	ND	Three key religious variables that have previously been demonstrated to relate with mental health in the Jewish community: Intrinsic motivations for religiosity, positive/negative religious coping, and trust/mistrust in God (Rosmarin et al., 2011a,b; Rosmarin et al., 2017; Pirutinsky and Rosmarin, 2018).
Pollock, D. M.	Religious	Process-based	Coping with catastrophe in the broadest sense of the word; encountering a stress or trauma and emerging as strong or stronger from the experience; the ability of a community to stick together and to help itself as a group as well as the families in its midst is an important coping behavior	“the ability of a community to stick together and to help itself as a group as well as the families in its midst”	Ganor M, Ben-Lavy Y. Community resilience: Lessons derived from Gilo under fire (2003).
Storr, V. H.; Haeffele-Balch, S.; Grube, L. E.	Geographical	Process-based	Recovery after natural catastrophe (hurricane). Being able to deal with profound uncertainty post-disaster	ND	Social learning by adapting existing organization structures and by creating new procedures and imitate the successful actions of others to spur recovery (Aldrich, 2012)
Storr, V. H.; Grube, L. E.; Haeffele-Balch, S.	Geographical	Process-based	How community members leverage social networks for mutual assistance, and in turn, contribute to community return and rebuilding. The ability of communities to engage in collective action and to overcome challenges; to organize and solve collective action problems is supported by strong social networks, shared values and past experiences with disaster.	ND	Theory of polycentric and monocentric orders, Elinor Ostrom (several), Coyne and Lemke (2012)
Vanhamel, J.; Meudec, M.; Van Landeghem, E.; Ronse, M.; Gryseels, C.; Reyniers, T.; Rotsaert, A.; Ddungu, C.; Manirankunda, L.; Katsuva, D.; Koen, P. G.; Nöstlinger, C.	Geographical	Process-based	Community resilience by fostering a feeling of being capable of collectively and successfully mitigating the COVID pandemic crisis like had been done in previous crises (Iron Curtain / World War II / Holocaust) and by self-organization and solidarity	ND	ND
Vollhardt, J. R.; Nair, R.	Geographical	Process-based	The group’s survival, success, and strength (Ferguson et al., 2010). This also reflects victim groups’ need for restored agency and empowerment (Shnabel and Nadler, 2015). Opposite to victimization, weakness, vulnerability, and struggle	ND	Dyadic, oppositional themata categories, which specific content may vary and change over time, based on the cultural and political context (Markova, 2015)

ND = No definition.

The reviewed literature was categorized according to the three previously reported perspectives on resilience, resource-based (Tengblad and Oudhuis, 2018), outcome-based and process-based (Infurna and Luthar, 2018; Wiles et al., 2019). In most of the articles, resilience was defined from a process-based perspective (*n* = 9), in six articles the resource-based perspective was leading, and the outcome-based perspective was identified in one article (Chalew, 2007). Chalew, describing the aftermath of Hurricane Katrina, also applies a process-based perspective as he distinguished three phases after the disaster: (1) saving lives (pikuach nefesh), (2) return to home and initial recovery, and (3) renaissance beyond recovery to transformation. Three articles (Glöckner, 2010; Frogel, 2015) combined a resource-based perspective with a process-based perspective. Even though these authors defined resilience as resource-based, all three articles describe processes of communities dealing with adversities.

### 3.3. Traumatic events or causes of stress described (review question 2)

The answer to review question 2 is listed in Table 1. The forms of adversity that were most studied were natural disasters, such as a flood (*n* = 3) and the COVID-19 pandemic (*n* = 3). Other adversities in the studies were (un)voluntary migration (*n* = 5) and five articles did not specify the adversity. Furthermore, eight of the sixteen articles (Carp, 2007; Heitlinger, 2009; Glöckner, 2010; Gidley and Kahn-Harris, 2012; Frogel, 2015; Vollhardt and Nair, 2018; Pirutinsky et al., 2020; Vanhamel et al., 2021) mentioned antisemitism as one of the adversities influencing the quality of life of the members of diasporic Jewish communities.

### 3.4. Factors facilitating community resilience (review question 3)

The inductive qualitative analysis identified that social and religious factors, strong organizations, and the role of education and communication were facilitating factors for community resilience. All articles describe the importance of good social networks within the community. Next, several articles mention the role of religion as a social factor to strengthen community resilience, and in the sense of a spiritual source of individual resilience. A good education was seen as a facilitator on an individual level, but Jewish educational institutions can also strengthen the community. Communication is important in dealing with adversity. Both articles about COVID-19 stress the role of the community in communication about the developments around the pandemic. This included digital and face-to-face communication, both within the community and from government institutions with community leaders (Aronson et al., 2020; Vanhamel et al., 2021). Elo and Vemuri (2016), Frogel (2015), and Pollock (2007) mentioned specific cultural narratives of resilience, grounded in the Jewish value system, norms, and identity, suggesting that these contribute to the willingness of Jews to invest in their community and the survival of Jewish diaspora communities. An active role in the community is rewarded by bringing societal status, respect, and satisfaction from the community to the individual (Elo and Jokela, 2014).

### 3.5. Barriers to community resilience (review question 4)

The qualitative analysis identified the following barriers to community resilience: The interaction with the hosting country and other communities, characteristics of the community itself, and psychological and cultural issues. Examples of the first group of barriers are legislation of the hosting country, antisemitism, and connection with Israel. An example of a characteristic of the community itself is the size of the community. Being a small community was considered by two authors as a disadvantage (Frogel, 2015; Elo and Vemuri, 2016). Examples of psychological issues were the impact of the Holocaust and a loss of identity. Cultural issues were different values between generations about family values or gender issues. Several authors also mentioned a language barrier that made it difficult to connect with sources outside the community or communicate between generations.

### 3.6. Comparing categorizations of community resilience

As a next step to gain further insight into the specific characteristics of the community resilience of the Jewish diaspora, all facilitators and barriers described in the included articles were deductively analyzed according to the elements of Patel et al. (2017). All articles included in this review described factors that fall in at least one of the nine main elements: local knowledge, community networks and relationships, communication, health, governance and leadership, resources, economic investment, preparedness, and mental outlook (Table 3). Furthermore, all main elements of Patel et al. were attributed more than once. The element ‘health’ was only found concerning mental health. The majority of studies on the resilience of the Jewish diaspora community described facilitators and barriers in the ‘economic investment,’ ‘social networks and relationships’ element.

**Table 3:** Factors for community resilience, categorized by the main elements of Patel et al.

	Local knowlegde	Community networks/relationships	Communication	Health	Governance/leadership	Resources	Economic investment	Preparedness	Mental outlook
Aronson, J.; Boxer, M.; Brookner, M. A.; Magidin de Kramer, R.; Saxe, L.				V			V		
Azoulay, B.; Sanchez, W.	V			V			V		
Carp, J. M.			V			V	V	V	
Chalew, G. N.					V	V	V		
Elo, M.; Vemuri, S.	V	V	V		V	V	V		V
Frogel, E.							V		V
Gidley, B., Kahn-Harris, K.					V				
Glöckner, O.		V	V				V		
Heitlinger, A.		V							
Horwitz, S.	V	V		V		V	V	V	
Pirutinsky, S.; Cherniak, A. D.; Rosmarin, D. H.				V					
Pollock, D. M.		V							V
Storr, V. H.; Haeffele-Balch, S.; Grube, L. E.	V				V				
Storr, V. H.; Grube, L. E.; Haeffele-Balch, S.	V	V					V		
Vanhamel, J.; Meudec, M.; Van Landeghem, E.; Ronse, M.; Gryseels, C.; Reyniers, T.; Rotsaert, A.; Ddungu, C.; Manirankunda, L.; Katsuva, D.; Koen, P. G.; Nöstlinger, C.		V	V		V	V			V
Vollhardt, J. R.; Nair, R.									V

A second framework was used for the further analysis of the community resilience characteristics of the Jewish diaspora, the seven categories of the Community Capital Framework of Flora et al. (2016) (Table 4). All retrieved facilitators and barriers in the sixteen included articles were analyzed using Atlas-ti. A total of 249 quotations were created, and all quotations were coded with one or more of the seven capitals of Flora and Flora. An eighth code was added by the researcher, called ‘Other.’ In all articles, social capital was nominated more than once, human capital was nominated in 13 out of 16 articles, cultural capital in 9 articles, and political and financial capital were both mentioned in 7 articles. Built capital was nominated in two articles and nature in one. In 5 articles characteristics were mentioned that were categorized as ‘other.’ The division of the total quotations is shown in Table 5.

**Table 4:** Factors for community resilience, categorized by the Community Capital Framework.

	Social	Human	Cultural	Political	Financial	Built	Natural	Other
Aronson, J.; Boxer, M.; Brookner, M. A.; Magidin de Kramer, R.; Saxe, L.	VV	VV	V	V	V		V	
Azoulay, B.; Sanchez, W.	VV	VV	VV	VV	VV			
Carp, J. M.	VV	VV						
Chalew, G. N.	VV				VV	VV		
Elo, M.; Vemuri, S.	VV	VV	VV	VV				V
Frogel, E.	VV	VV	VV	VV				
Gidley, B.; Kahn-Harris, K.	VV	VV						
Glöckner, O.	VV	VV	V	V	VV			
Heitlinger, A.	VV	VV	V	V				V
Horwitz, S.	VV	VV			V			VV
Pirutinsky, S.; Cherniak, A. D.; Rosmarin, D. H.	VV	VV			V			V
Pollock, D. M.	VV		VV					
Storr, V. H.; Haeffele-Balch, S.; Grube, L. E.	VV							
Storr, V. H.; Grube, L. E.; Haeffele-Balch, S.	VV	V		V	VV	V		V
Vanhamel, J.; Meudec, M.; Van Landeghem, E.; Ronse, M.; Gryseels, C.; Reyniers, T.; Rotsaert, A.; Ddungu, C.; Manirankunda, L.; Katsuva, D.; Koen, P. G.; Nöstlinger, C.	VV	VV	V					
Vollhardt, J. R.; Nair, R.	VV	VV	V					

0, not mentioned; V, mentioned once; VV, mentioned more then once.

**Table 5:** The factors, divided over the seven community capitals of Flora and Flora.

Community capitals (Flora and Flora)	Numbers
Social capital	127
Human capital	62
Cultural capital	20
Political capital	16
Financial capital	12
Built capital	4
Natural capital	1
Other	6
Total	249

As shown in Table 5, the most frequently mentioned positive factor in the reviewed articles was related to social capital. Many social factors were mentioned by more than one author, such as the role of the family, marriage, friendship and the community, the role of other Jewish communities, the hosting country, the country they left, or Israel. Factors related to human capital were also mentioned often, such as language skills or psychological issues. The importance of the ‘sense of belonging’ was mentioned several times. Cultural and political factors came in third and fourth place. Financial factors were mentioned both on an individual level – the risk that people’s incomes drop after adversity – and on a community level – thus the need for funding after a disaster. Factors relating to the built capital were only mentioned four times, always related to a disaster, and natural capital was mentioned only once, indirectly related to people and their interests during COVID-19. The code ‘other’ was applied for two types of factors: several quotations regarding the availability of technology and the internet for information and social connection, and one for the factor ‘time’ (Elo and Vemuri, 2016). A few authors claim there is a direct relationship between one of the factors and people’s involvement in a resilient community. Glöckner (2010) cites Cohen stating that ‘increasing economic security in private life normally correlates with a growing commitment in the local Jewish community.’

It was observed that the articles on natural disasters refer more often to the material categories, except for one article by Storr that deals specifically with social capital (Storr et al., 2016). Articles dealing with more continuous adversities tend to focus on the immaterial categories. Some authors (Pollock, 2007; Frogel, 2015; Elo and Vemuri, 2016) suggest that Jewish diaspora communities tend to be more resilient due to specific factors, such as Jewish values or being part of a religious community and are thus more capable to deal with adversities.

Previously, it has been suggested that culture and changing environments due to migration and living in the diaspora may significantly impact resilience (Ungar, 2008). Two studies that have reported on the resilience of Jewish communities in the USA involve the way they respond to disasters, specifically, Hurricanes Katrina and Sandy (Chalew, 2007; Storr et al., 2017). Storr et al. (2017) found that privately organized social service providers within the community that joined to coordinate their actions were a key factor in the recovery of the Jewish community in New York after Hurricane Sandy. Chalew (2007) described the rise of a cohesive, committed, and revitalized Jewish community two years after Hurricane Katrina. The key factors for this transformation were found to be the financial generosity of the American Jewish community as a whole and the community leaders who acted to build a new future for the community after the disaster. A recently published study that reported on the importance of the community for the resilience of its members investigated the response of the Antwerp Jewish communities to the COVID-19 pandemic (Vanhamel et al., 2021). In this study, the importance of engaging communities and religious leaders in risk communication and local decision-making was significant in dealing with pandemic control measures and the impact of COVID-19.

### 3.7. Methods to measure community resilience (review question 5)

The resilience of the Jewish diasporic communities was mainly investigated and narratively described by qualitative methods: (semi-structured) interviews, focus groups, case studies, (field) observations, or an analysis of data from historical, ethnographic, and strategic documents. None of the articles included quantitatively measured community resilience. One article by Aronson et al. (2020) surveyed aspects of individual resilience and indicated the level of involvement in a Jewish community.

### 3.8. The relationship between community resilience and the health-related quality of life of individuals (review question 6)

Flora et al. (2016) expect that the capitals support sustainability, consisting of a healthy ecosystem, social inclusion, and economic security, which in turn form the quality of life (Vaneeckhaute et al., 2017). None of the articles referred explicitly to the concept of quality of life, in relationship with the resilience of the community, nor used measuring methods and outcomes regarding the quality of life of members of the community.

### 3.9. Key gaps in the literature on community resilience (review question 7)

The findings of this scoping review reveal several key gaps in the literature on the resilience of Jewish diasporic communities. No univocal definition was found, nor methods to measure community resilience, nor was there elaboration on what community resilience meant for the quality of life of individuals.

Only a few articles were based on theoretical modeling or a framework, and just one author (Pollock, 2007) refers to a theoretical model of community resilience developed by Ganor and Ben-Lavy (2003). Even though nine articles were based on empirical research methods, only one study applied triangulation (Vanhamel et al., 2021), no mixed-methods research was conducted, and, in most studies, the number of participants was small. The majority of studies focused on one type of adversity, a disaster or COVID-19, whereby a more overall perspective of resilience was lacking.

### 3.10. Ethical issues or challenges studying community resilience (review question 8)

Two authors questioned their role as researchers, one because the researcher was not part of the studied community (Vanhamel et al., 2021), or – on the contrary – because the researcher was part of the studied community (Frogel, 2015). Vanhamel et al. (2021) mentioned that due to his position as a researcher from outside the community he was not able to explore in more depth some social and cultural issues within the Jewish community toward the Belgian government and community. Frogel (2015) wonders if it might have influenced the interviews but indicates mostly positive aspects of her being part of the community. Vollhardt and Nair (2018) report the possibility of socially desirable answers due to group dynamics within the focus groups. They also describe the way they prepared and debriefed the respondents, given the sensitive nature of the topic.

As Jewish identity is not synonymous with the Jewish religion, there are many forms of Jewish identity. The issue of different and variable self-identifications was found in the study on the Afghan Jewish community (Elo and Vemuri, 2016) and the article of Azoulay (Azoulay and Sanchez, 2000). The study of Gidley and Kahn-Harris (2012) also mentions that the Anglo-Jewish community is an entity in motion. Instead, they use the terms “messy, contingent, fluid, evolving and contested” (p. 183, Gidley and Kahn-Harris, 2012) to describe the Jewish diaspora community. Gidley and Kahn-Harris point out another ethical challenge that relates to the neutrality of the researcher. According to them, Jewish leadership has used the existence of antisemitism to regain leadership. The wording they use by describing this reveals a judgment on the behavior of Jewish leadership doing so.

## 4. Discussion

To the best of our knowledge, this scoping review is the first comprehensive systematic review focusing on community resilience in the Jewish diaspora community. This review found sixteen studies targeting the concepts of the resilience of the Jewish communities in the diaspora and identified several key factors affecting the resilience of these communities.

### 4.1. Discussion of major findings in relation to existing literature

The present scoping review reveals that community resilience is mostly described in terms of coping with disaster or struggling with acculturation of the Jewish diaspora. Most articles described concepts that were closely related to resilience, such as adaptation, acculturation, or coping, as were the concepts of trauma and posttraumatic stress disorder (PTSD). While these concepts highlight the individuals’ reactions to impactful events, the concept of resilience can be applied to both individual and community responses to such events. A clear definition of community resilience of the Jewish diaspora as such, thus seems to be lacking in the literature. Only one article defined the concept of community resilience and associated it with the theoretical model of Ganor and Ben-Lavy (2003) and Pollock (2007).

In the present scoping review we found that in most studies (*n* = 9) the Jewish diaspora communities were characterized as a place-based community (Chalew, 2007; Storr et al., 2016, 2017). Place-based communities are communities of people who are bound together because of where they reside, work, visit or otherwise spend a continuous portion of their time (Gieryn, 2000). This finding is well in line with other studies on community resilience that mainly involved place-based communities (Kulig et al., 2013; Flora et al., 2016; Vaneeckhaute et al., 2017). The other studies included in this review characterized the Jewish diaspora communities by the country of origin of the members, or by religious affiliation. It thus appears from this scoping review that the Jewish diaspora communities world-wide differ from one another regarding their specific geographic location, migration history and religious affiliation. It is well-known from the literature that the migration history of Jews, especially in Europe, is ancient (Blom et al., 2021) and that in many countries, the Jewish diaspora communities are prosperous in the socioeconomic sense (Wallerstein and Duran, 2010). In contrast to this, we were reminded in the present study that Jewish diaspora communities have been established in the last decennia (Elo and Vemuri, 2016) or consist of members with a more marginalized position (Glöckner, 2010). Jewish diaspora communities appear to have some characteristics which make them different from most studied diaspora communities; they have variously been considered a race, an ethnic group, members of a religion, or a culture. These characteristics lay the foundation of an ongoing scientific debate on the question if Jews can be considered an ethnic minority. However, a comparison of the results of this scoping review with those of Patel et al. (2017) and Flora et al. (2016) suggest that many notions of community resilience are also applicable to the studies on Jewish diaspora communities.

Another important finding of this scoping review is that all three perspectives on resilience – resource-based, outcome-based, and process-based – are found in the research on Jewish diaspora communities world-wide. The process-based perspective appears to be the most common perspective on resilience of Jewish communities living in the diaspora. This finding is in line with the results of Patel et al. (2017), who also demonstrated that the process-based perspective was adopted in most recent studies on resilience of other (non-Jewish) communities. This could be explained by the fact that the context of Jewish diaspora communities worldwide varies substantially, and that this impacts their resilience resources. As both the resources and the adversities can vary, also the outcomes vary. Therefore, research from a process-based perspective appears to be the most relevant when searching for generalizable ways to strengthen the resilience of Jewish diaspora communities.

In the general literature on community resilience, numerous studies and policy documents address the distinct phases communities go through when dealing with adversities and distinguish four phases of dealing with a disaster: preparedness, mitigation, response, and recovery (Maguire and Hagan, 2007; FEMA, 2019). In this scoping review comparable phases were identified in articles that describe the aftermath of a natural disaster (Chalew, 2007; Storr et al., 2016, 2017) and in the policy-oriented articles (Carp, 2007; Horwitz, 2007; Pollock, 2007).

Through this scoping review, we identified that social factors and networks are strong positive influencing factors for the resilience of Jewish diaspora communities. These enablers of community resilience have also been reported by others. Based on interviews with Australian rural community members, Buikstra et al. (2010) reported the presence of social networks and support as a critical factor for community resilience. Faulkner et al. (2018) conducted an empirical review of five interrelated characteristics of community resilience, of which one was community networks. They reported that the residents of two different coastal communities in the UK perceived these characteristics in different combinations of importance for enabling resilience. It was concluded that context is important, that context consists of many factors which are interconnected [e.g., past experiences with crisis events, and that strengthening any one factor in isolation from others will probably not lead to enhanced levels of community resilience (Faulkner et al., 2018)]. Based on a group of survivors of an earthquake in China, Wei et al. (2022) reported recently that social capital is the most consistent and positive predictor of perceived community resilience. Liu analyzed the impact of social networks on community resilience in Tianjin (China) and pointed to social trust as a core element, as trust affects the willingness to be involved in communities’ activities and networks (Liu et al., 2022).

The influencing factors for community resilience that were identified in this scoping review were categorized and analyzed using the list of the nine main elements of Patel et al. (2017) and the seven capitals of the Community Capital Framework of Flora et al. (2016). Both classifications are referred to in many studies and have contributed to the understanding of community resilience. They are based on different perspectives on adversities, one-time disasters, and ongoing stress, respectively. The classifications partly overlap, and partly highlight different elements. Further research is needed to find out how these classifications exactly relate to each other. In addition, it is important to highlight that in our analysis we identified two other influencing factors ‘time’ and ‘technology and internet,’ that could not be classified in any of the categories of Flora and Flora. Patel et al. (2017) mentions technology and social media as a sub-category of the element ‘communication.’ Adding the factors ‘time’ and ‘technology and internet’ as separate categories to the classifications might thus be relevant for an overall contemporary perspective on community resilience.

### 4.2. Strengths and limitations

#### 4.2.1. Strengths

A rigorous and systematic methodology was applied in the present scoping review. The search strategy was adapted as broadly as possible in databases without controlled vocabularies to obtain the maximum search yield. In addition to the database searches, gray literature was searched. Furthermore, two independent researchers screened and included all articles. Another strength of this review is that it was conducted by a multidisciplinary team with researchers from different backgrounds including public health, philosophy, psychiatry, and psychology. The fact that the research questions were intensively discussed with three members of Dutch and Israeli Jewish communities can also be considered a strength of this study.

#### 4.2.2. Limitations

Although 32% of the Jews currently living in Israel are migrants, many Jews worldwide consider Israel as their home country and regard the Jewish community in Israel as the dominant community and not a diaspora community. Because we specifically aimed to explore the characteristics of the Jewish diasporic community outside their home country, where they are a minority, a considerable number of studies on communities in Israel were thus excluded from this scoping review. Some of these excluded Israeli studies (Leykin et al., 2013; Kimhi, 2016; Leykin et al., 2016; Shapira, 2022; Weinberg and Kimchy Elimellech, 2022) may have been relevant for the Jewish diaspora communities. Kimhi, for example, studied the association between individual, community and national resilience and found significant positive but low correlations between community and individual resilience (*R* = 0.160). Based on that, Kimhi assumes that each resilience level stands for an independent construction, but both predict individual well-being and successful coping with potentially traumatic events (Kimhi, 2016). Shapira and Leykin conducted some of the few longitudinal studies on community resilience in Israeli communities in response to several adversities. Shapira focused on how perceived community resilience levels change over time and while dealing with different hazards (Shapira, 2022). They concluded that throughout the study period, place attachment, collective efficacy, and preparedness were the strongest contributors to community resilience, while trust in local leadership and social trust were the weakest. Shapira’s study also confirms the notion that different adversities impact psychological demands on exposed populations differently, and in turn, affect coping strategies and resiliency. Leykin and Cohen developed the Conjoint Community Resiliency Assessment Measure (CCRAM), a validated instrument to measure community resilience (Cohen et al., 2013; Leykin et al., 2013). Leykin compared community resilience during emergency and routine situations using the CCRAM and confirmed that during an emergency, higher resilience trends will emerge (Leykin et al., 2016). Only one of the five community resilience factors, social trust, stayed constant over time. Weinberg examined the effect of spirituality and perceived community resilience on PTSD and stress of first responders and showed that spirituality, age, and financial situation were negatively associated with PTSD symptoms and stress. However, perceived community resilience was not associated with PTSD symptoms or stress (Weinberg and Kimchy Elimellech, 2022). Summarizing, these insights and instruments are relevant and can contribute to understanding the resilience of Jewish diaspora communities. Another limitation of the present scoping review is that the majority of the reviewed articles were on Jewish diaspora communities living in the United States of America (USA). Therefore, results need to be interpreted with caution and cannot be extrapolated to Jewish diaspora communities worldwide. More research is needed in other countries, specifically because the exploration of the role of history, culture, religion, ethnicity, and antisemitism in and outside the USA may be critical in understanding community resilience and the impact on the individual resilience and health of their members. Lastly, as community resilience is not a well-defined concept (Kulig et al., 2013; Patel et al., 2017), it was sometimes up to the personal understanding and interpretation of the authors of this scoping review whether the article dealt with community resilience of the Jewish diaspora. For this reason, some relevant articles or concepts may have been missed.

### 4.3. Recommendation for practice and further research

As mentioned in the introduction, most Jews worldwide live in the diaspora (The Jewish Agency for Israel, 2022). There are constant migrant movements of Jews, sometimes as refugees, due to wars or antisemitism, and sometimes as regular migrants looking for a better life. One of the most recent Jewish migrant movements is the migration from Ukraine (McKernan and Kierszenbaum, 2022; Schut, 2022). Because of the constant migrant movements, Jewish diaspora communities are very diversified. Jewish history is a rich history of constantly investing in and (re-)building their communities. Recently, several programs have been developed (Gidron, 2019; Baker, 2020) aiming to strengthen Jewish community resilience within and outside Israel. Our findings in the present scoping review on facilitators and barriers for community resilience are therefore of relevance for these programs and the future support and development of Jewish diaspora communities.

Key gaps in the literature that were identified in this scoping review are that studies on community resilience of Jewish diaspora communities focused on a single crisis only and that none of the included studies applied assessment measures. In addition, no study looked at the association between individual and community resilience, nor described how community resilience affects the health-related quality of life of individuals in this diasporic population. The WHO Health Evidence Network conducted a review of methods to measure health-related community resilience (South et al., 2018) in which different research methods to assess community resilience were evaluated. This study concluded that health-related community resilience is a complex, multidimensional concept. Therefore, research methods should collect data from multiple domains, prioritize social and economic indicators and intersectoral cooperation, combine quantitative and qualitative data or apply a mixed-methods research strategy (South et al., 2018). Infurna and Luthar (2018) and Infurna and Jayawickreme (2019) also show that resilience is a multidimensional construct and expresses the need for a more comprehensive theory and a thorough multidimensional research approach.

Research on community resilience can broadly be divided into two approaches. The first approach studies the collective resilience *of* communities, whereas the second approach studies the individual resilience of people *in* communities. Van der Schoor (2020, unpublished, see footnote 1) concluded that hardly any research has been conducted on the interactions between these perspectives. Further research on the lived experiences of members of Jewish diaspora communities can contribute to the understanding of these interactions. Pearson and Geronimus (2011) found that the self-rated health of Jewish Americans was significantly worse than that of other White Americans, and access to co-ethnic social ties was associated with better self-rated health among Jews. None of the reviewed articles in the present study mentioned any relationship between the resilience of Jewish diasporic communities and the health-related quality of life of individuals belonging to the community. In her study on Afghan Jews and their children, Fogel distinguishes eight themes and relates them to the ecological model of Bronfenbrenner (Frogel, 2015). However, she does not analyze the dynamics between these levels. This gap needs further research.

For a better understanding of social support, generally considered to be one of the main contributors to individual resilience, it is recommended that future studies reporting on community resilience focus on the interactions between Jewish diaspora communities and their members, and other diaspora communities.

### 4.4. Conclusion

Social and religious factors, strong organizations, education, and communication were identified as facilitating factors that increase community resilience. The social factors mentioned were marriage, the family and other social networks, and a sense of belonging and social connections. Religious factors were religious traditions, identity and coping and the role of religious gatherings. The interaction with the hosting country and other communities, characteristics of the community itself, and psychological and cultural issues were specified barriers to the resilience of Jewish communities in the diaspora. The effect of antisemitism in the hosting country as a barrier was mentioned in half of the articles. Community characteristics were its small size and or a lack of unity and dividedness.

The results of this study contribute to a better understanding of the meaning of community resilience and the facilitating factors and barriers for Jewish diasporic communities and their members. Research examining the relevance and importance of resilience in the context of diaspora communities is still lacking. A better understanding of the resilience of Jewish diaspora communities can contribute to strengthen the resilience of Jewish and other diaspora communities. Thus, further research on Jewish communities can help Jewish and other diasporic communities, their leaders, policymakers and supporting organizations to strengthen the resilience of these communities and their members to deal with future traumatic stress.

## Data availability statement

The original contributions presented in the study are included in the article/Supplementary material, further inquiries can be directed to the corresponding author.

## Author contributions

JM, AM, GS, and MJ: conceptualization and formal analysis. JM, WS, and MJ: data curation. WS and JL: searches. JM and MJ: screening and inclusion. JM and WS: data extraction. JM: project administration and writing – original draft. MJ: validation. MJ, AM, GS, and JM: writing – review & editing. All authors contributed to the article and approved the submitted version.

## Conflict of interest

The authors declare that the research was conducted in the absence of any commercial or financial relationships that could be construed as a potential conflict of interest.

## Publisher’s note

All claims expressed in this article are solely those of the authors and do not necessarily represent those of their affiliated organizations, or those of the publisher, the editors and the reviewers. Any product that may be evaluated in this article, or claim that may be made by its manufacturer, is not guaranteed or endorsed by the publisher.

## Footnotes

^1^Van der Schoor, Y. (2020). The impact of local resources on CR. Unpublished.
