# Supplementary material for: The resilience of Jewish communities living in the diaspora: a scoping review
Source: Front Psychol. 2023 Aug 16;14:1215404. doi: 10.3389/fpsyg.2023.1215404 (PMC10466794; doi:10.3389/fpsyg.2023.1215404)
Supplement: Supplementary file 2 [file Data_Sheet_2.docx]

**S2: Search strategy**

**PsycInfo**

Population: Jewish communities outside Israel

(exp judaism/ or exp Jews/ OR  AntiSemitism/ OR Holocaust/ or Holocaust Survivors/ OR Concentration Camps/ OR (jew* or judai* or Ashkenazi* OR Sephardi* OR holocaust or antisemitis* OR (anti ADJ1 semitis*) OR ((concentration or kz) ADJ1 camp*)).mp.)

Concept: Resilience

(exp "Resilience (Psychological)"/ OR (resilien* or hardiness OR endur* OR adapt* OR grit* OR recover* OR rehabilit* OR ((Emotional OR Psychological OR mental* OR behavio*) ADJ3 (Adjust* OR stable OR Stabilit* OR flexib* OR tough* or strength* OR weak*)) or ((Posttraumatic OR post-trauma*) ADJ3 (Growth)) OR (( deal* OR handl* OR sensiti* OR coping OR cope) ADJ3 (distress* OR stress* or advers* OR trauma* OR event* OR experience* OR difficult* OR challen* OR demand***)) OR ((positive) ADJ3 (health* OR psycholog*)) OR ((protect*) ADJ3 (factor*)) ).mp.)

Context: Diaspora communities

(exp Human Migration/ or exp Ethnic Identity/ or exp Social Identity/ or exp Communities/OR Immigration/ OR (Diaspora or communit*, exodus, emigrant*, emigrat* OR migrant* OR migrat* OR immigrant* OR immigrat* or transient* or movement* or mobility OR relocat* or exile* or expat* or settler* OR refugee* OR asylumseek* OR ((asylum) ADJ3 (seek* OR political)) OR ((displaced) ADJ3 (person*)) OR identit*).mp.)

1 and 2 and 3

**Medline ALL**

Population: Jewish communities outside Israel

(exp judaism/ or exp Jews/ OR  Holocaust/ or Concentration Camps/ OR (jew* or judai* or Ashkenazi* OR Sephardi* OR holocaust or antisemitis* OR (anti ADJ1 semitis*) OR ((concentration or kz) ADJ1 camp*)).mp.)

Concept: Resilience

(exp " Resilience, Psychological"/ OR (resilien* or hardiness OR endur* OR adapt* OR grit* OR recover* OR rehabilit* OR ((Emotional OR Psychological OR mental* OR behavio*) ADJ3 (Adjust* OR stable OR Stabilit* OR flexib* OR tough* or strength* OR weak*)) or ((Posttraumatic OR post-trauma*) ADJ3 (Growth)) OR (( deal* OR handl* OR sensiti* OR coping OR cope) ADJ3 (distress* OR stress* or advers* OR trauma* OR event* OR experience* OR difficult* OR challen* OR demand***)) OR ((positive) ADJ3 (health* OR psycholog*)) OR ((protect*) ADJ3 (factor*)) ).mp.)

Context: Diaspora communities

(exp Human Migration/ or exp Social Identification/ or exp Population Groups / OR (Diaspora or communit* or exodus or emigrant* or emigrat* OR migrant* OR migrat* OR immigrant* OR immigrat* or transient* or movement* or mobility OR relocat*, exile*, expat*, or settler* OR refugee* OR asylumseek* OR ((asylum) ADJ3 (seek* OR political)) OR ((displaced) ADJ3 (person*)) OR identit*).mp.)

1 and 2 and 3

**Embase**

Population: Jewish communities outside Israel

(exp judaism/ or exp Jew/ OR Holocaust/ or Concentration Camp/ OR (jew* or judai* or Ashkenazi* OR Sephardi* OR holocaust or antisemitis* OR (anti ADJ1 semitis*) OR ((concentration or kz) ADJ1 camp*)).mp.)

Concept: Resilience

(exp psychological resilience/OR (resilien* or hardiness OR endur* OR adapt* OR grit* OR recover* OR rehabilit* OR ((Emotional OR Psychological OR mental* OR behavio*) ADJ3 (Adjusted * OR stable OR Stabilit* OR flexib* OR tough* or strength* OR weak*)) or ((Posttraumatic OR post-trauma*) ADJ3 (Growth)) OR (( deal* OR handl* OR sensiti* OR coping OR cope) ADJ3 (distress* OR stress* or advers* OR trauma* OR event* OR experience* OR difficult* OR challen* OR demand***)) OR ((positive) ADJ3 (health* OR psycholog*)) OR ((protect*) ADJ3 (factor*)) ).mp.)

Context: Diaspora communities

(exp Migration/ or exp Identity/ or exp community/ OR (Diaspora or communit*, exodus, emigrant*, emigrat* OR migrant* OR migrat* OR immigrant* OR immigrat* or transient* or movement* or mobility OR relocat*, exile*, expat* or settler* OR refugee* OR asylumseek* OR ((asylum) ADJ3 (seek* OR political)) OR ((displaced) ADJ3 (person*)) OR identit*).mp.)

1 and 2 and 3

**PTSDpubs**

Population: Jewish communities outside Israel

MAINSUBJECT.EXACT("Jews") OR MAINSUBJECT.EXACT("Holocaust") OR MAINSUBJECT.EXACT("Concentration Camps") OR TI, AB, SU, TM (jew* or judai* or Ashkenazi* OR Sephardi* OR holocaust or antisemitis* OR (anti NEAR/1 semitis*) OR ((concentration or kz) NEAR/1 camp*))

Concept: Resilience

MAINSUBJECT.EXACT("Resilience") OR MAINSUBJECT.EXACT("Community Resilience")

Context: Diaspora communities

MAINSUBJECT.EXACT. EXPLODE("Migration") OR MAINSUBJECT.EXACT("Ethnic Identity") OR TI, AB, SU, TM (Diaspora or communit* or exodus or emigrant* or emigrat* OR migrant* OR migrat* OR immigrant* OR immigrat* or transient* or movement* or mobility OR relocat* or exile* or expat* or settler* OR refugee* OR asylumseek* OR (asylum NEAR/3 (seek* OR political)) OR (displaced NEAR/3 person*) OR identit*)

1 and 2 and 3

SSRN

Zie werkblad.xls

Opengrey.com

 (jew* or judai* or Ashkenazi* OR Sephardi* OR holocaust or antisemitis* OR (anti NEAR/1 semitis*) OR ((concentration or kz) NEAR/1 camp*) ) AND (resilien* or hardiness OR endur* OR adapt* OR grit* OR recover* OR rehabilit* OR ((Emotional OR Psychological OR mental* OR behavio*) NEAR/3 (Adjust* OR stable OR Stabilit* OR flexib* OR tough* or strength* OR weak*)) or ((Posttraumatic OR post-trauma*) NEAR/3 (Growth)) OR (( deal* OR handl* OR sensiti* OR coping OR cope) NEAR/3 (distress* OR stress* or advers* OR trauma* OR event* OR experience* OR difficult* OR challen* OR demand***)) OR ((positive) NEAR/3 (health* OR psycholog*)) OR ((protect*) NEAR/3 (factor*)) ) AND (Diaspora or communit* or exodus or emigrant* or emigrat* OR migrant* OR migrat* OR immigrant* OR immigrat* or transient* or movement* or mobility OR relocat* or exile* or expat* or settler* OR refugee* OR asylumseek* OR (asylum NEAR/3 (seek* OR political)) OR (displaced NEAR/3 person*) OR identit*)

**Sociological Abstracts**

Population: Jewish communities outside Israel

MAINSUBJECT.EXACT. EXPLODE("Jews") OR MAINSUBJECT.EXACT. EXPLODE("Judaism") OR MAINSUBJECT.EXACT("Anti-Semitism") OR MAINSUBJECT.EXACT("Holocaust") OR MAINSUBJECT.EXACT("Concentration Camps") OR TI, AB, SU, TM (jew* or judai* or Ashkenazi* OR Sephardi* OR holocaust or antisemitis* OR (anti NEAR/1 semitis*) OR ((concentration or kz) NEAR/1 camp*))

Concept: Resilience

MAINSUBJECT.EXACT("Resilience") OR MAINSUBJECT.EXACT("Community Resilience")

Context: Diaspora communities

MAINSUBJECT.EXACT.EXPLODE("Migration") OR MAINSUBJECT.EXACT.EXPLODE("Migration Patterns")OR MAINSUBJECT.EXACT.EXPLODE("Cultural Identity") OR MAINSUBJECT.EXACT.EXPLODE("Social Identity") OR MAINSUBJECT.EXACT.EXPLODE("Ethnic Identity") OR MAINSUBJECT.EXACT.EXPLODE("Group Identity") OR MAINSUBJECT.EXACT.EXPLODE("Communities") OR TI,AB,SU,TM(Diaspora or communit* or exodus or emigrant* or emigrat* OR migrant* OR migrat* OR immigrant* OR immigrat* or transient* or movement* or mobility OR relocat* or exile* or expat* or settler* OR refugee* OR asylumseek* OR (asylum NEAR/3 (seek* OR political)) OR (displaced NEAR/3 person*) OR identit*)

1 and 2 and 3
